# Unified risk analysis in radiation therapy

**DOI:** 10.1016/j.zemedi.2022.08.006

**Published:** 2022-10-07

**Authors:** Daniel Lohmann, Maya Shariff, Philipp Schubert, Tim Oliver Sauer, Rainer Fietkau, Christoph Bert

**Affiliations:** Department of Radiation Oncology, Universitätsklinikum Erlangen, Friedrich-Alexander-Universität Erlangen-Nürnberg, Universitätsstraße 27, 91054 Erlangen, Germany; Comprehensive Cancer Center Erlangen-EMN (CCC ER-EMN), Erlangen, Germany

**Keywords:** Risk analysis, UML, Activity diagram, IEC 80001-1

## Abstract

**Purpose:**

The increasing complexity of new treatment methods as well as the Information Technology (IT) infrastructure within radiotherapy require new methods for risk analysis. This work presents a methodology on how to model the treatment process of radiotherapy in different levels. This subdivision makes it possible to perform workflow-specific risk analysis and to assess the impact of IT risks on the overall treatment workflow.

**Methods:**

A Unified Modeling Language (UML) activity diagram is used to model the workflows. The subdivision of the workflows into different levels is done with the help of swim lanes. The model created in this way is exported in an xml-compatible format and stored in a database with the help of a Python program.

**Results:**

Based on an existing risk analysis, the workflows CT Appointment, Glioblastoma Multiforme, and Deep Inspiration Breath Hold (DIBH) were modeled in detail. Part of the analysis are automatically generated workflow-specific risk matrices including risks of medical devices incorporated into a specific workflow. In addition, SQL queries allow to quickly retrieve e.g., the details of the medical device network installed in a department.

**Conclusion:**

Activity diagrams of UML can be used to model workflows in radiotherapy. Through this, a connection between the different levels of the entire workflow can be established and workflow-specific risk analysis is possible.

## Introduction

Radiation therapy is technically one of the most complex medical treatment methods. Various technical and organizational steps are necessary to complete the treatment involving a variety of equipment operated by different professional groups. The treatment usually extends over several weeks from admission to discharge. An error that creeps in at the beginning of the treatment chain can, if not discovered early on, continue until the radiation treatment at the linear accelerator. Radiation therapy is also very dependent on functioning IT, so it is important for a risk analysis to also consider potential failure modes of the IT infrastructure. One specific issue in this respect is the medical device network since in recent years the number of devices with a network connection has increased steadily [1].

A risk analysis concept for interconnected medical devices is described by the IEC 80001-1 [2]. The document includes the protection goals of safety, effectiveness, and security. A violation of one of these protection goals can mean a risk for the patient or the treatment process.

Although not mandatory for all hospitals, the Euratom Directive 2013/59 demands a comprehensive risk analysis before introducing new treatment methods and when changing an existing treatment method.

Two widely used methods to perform risk analysis are Failure Mode and Effect Analysis (FMEA) and Fault Tree Analysis (FTA). The FMEA is used to prevent errors in complex systems that occur in day-to-day operations in advance. The FTA is downstream of the FMEA and creates a fault tree for each failure mode stated in the FMEA. There is already extensive literature describing the methodology in general as well as for radiation oncology in particular [4], [5], [6], [7], [8], [9]. To benchmark an FMEA, incident learning systems can be used, which also serve as in input of risk management in a retrospective manner [10], [11], [12], [13], [14], [15]. In radiation therapy, Ford et al. attempted to implement an FMEA-based approach to improve the treatment quality [5]. This includes setting up incident learning systems as well as implementing risk analysis group meetings. Although there are publications that take into account the technical aspect of care delivery, the previous variants of FMEA for radiation oncology clinical use focus mainly on the human factor within the process chain, which seems to be responsible for most of the errors within the therapy process [22].

Typically, for this purpose, table structured failure modes of an FMEA often combined with graphical representations of the process flow are used.

There is also already a publication that describes graphical modeling of a workflow in the form of an activity diagram as a directed graph, which takes different process levels into account [17]. Munbodh et al. describes the risks and workflow steps that occur during the pretreatment physics chart review (TPCR). Activity diagrams can be used to model a radiotherapy workflow as a graph. This is a diagram type from the Unified Modeling Language (UML), which is used primarily in software development [18], [19]. Activities are the individual workflow steps. A workflow results by connecting the workflow steps.

To our knowledge, there is no description of a model that describes the entire treatment process with different workflows and associated risks as well as the IT level. By using activity diagrams, a procedure is to be presented that represents such a model. As a result, one can track and assess the risks of the entire treatment chain, from the admission to discharge of the patient, in one model including the medical devices which support the overall workflow.

## Materials and methods

### Notation

**Figure 1:**
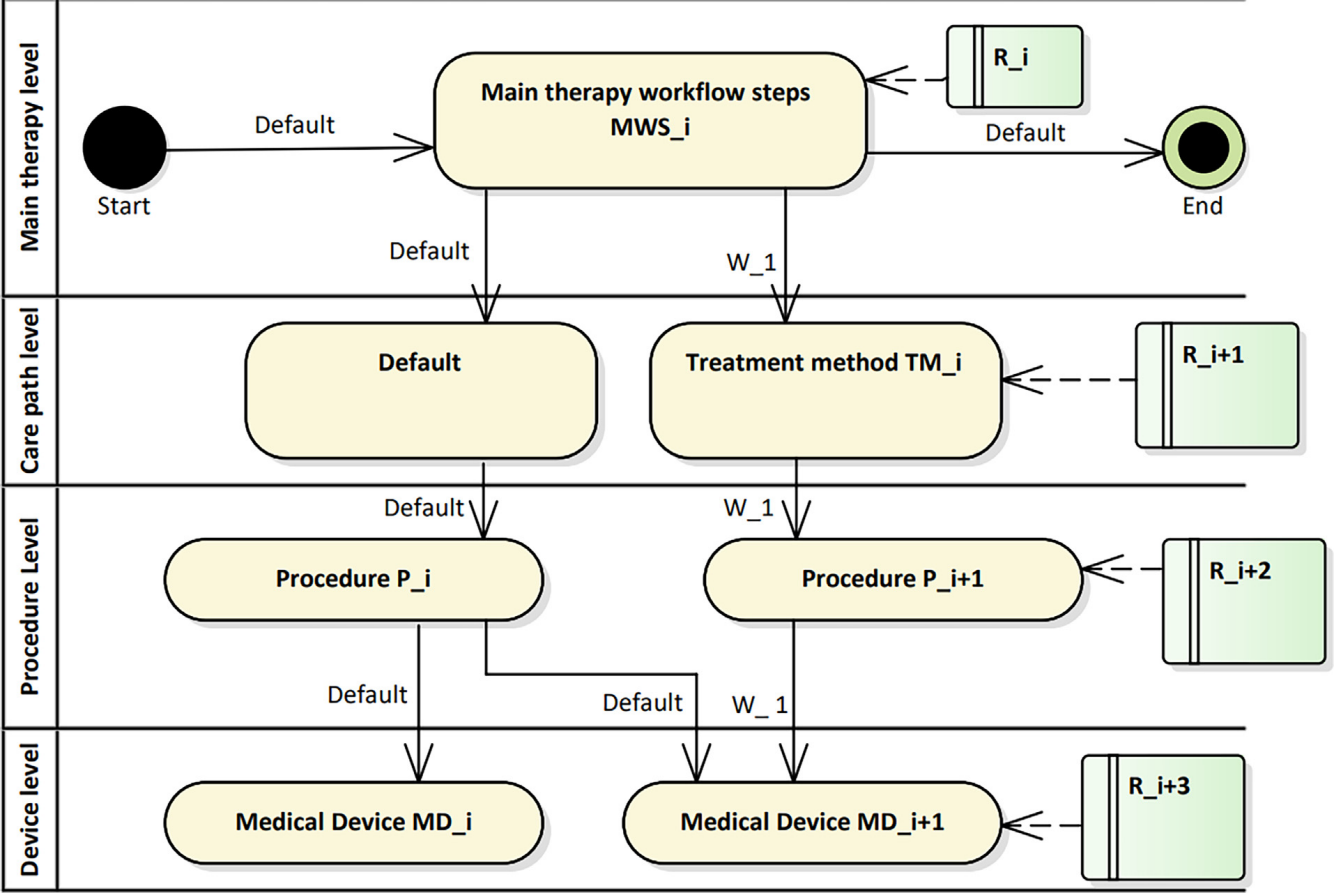
Workflows are represented by arrows and labeled with their name above. The swim lanes on the left represent the workflow levels. The yellow boxes are activities that can take different forms. The green boxes are requirements that take the form of risks. Each workflow has a start and an end point. ${\mathrm{MWS}}_{i}$, $P_{i}$, etc. are unique identifiers that e.g. allow using an activity or requirement in multiple workflows. Note, that once the workflow reaches the deepest level of the nested diagram it recursively returns to the next higher activity.

To represent a radiotherapy workflow in UML, an activity diagram can be used as shown in Fig. 1. Such a diagram consists of activities (yellow boxes) and arrows defining a workflow that runs recursively from start to end. In addition, requirements (green boxes) can be linked to individual activities. Activity diagrams can be created with UML-capable software assigning a unique identifier to each element. In our case Enterprise Architect (v15.2, SparxSystems) has been used.

For better structuring, activities and thus workflows can be organized in partitions or Swim-Lanes (also called Rummler-Brache diagram [20]). A workflow is indicated by a sequence of arrows $W_{i}=\left\{W_{1},W_{2},\ldots \right\}$ as well as the default workflow, which will be described later. The workflows are traversed from top to bottom in a recursive manner and then from left to right.

In the context of radiation oncology, we currently defined four swim lanes as main therapy level, care path level, procedure level, and device level.

**Figure 2:**
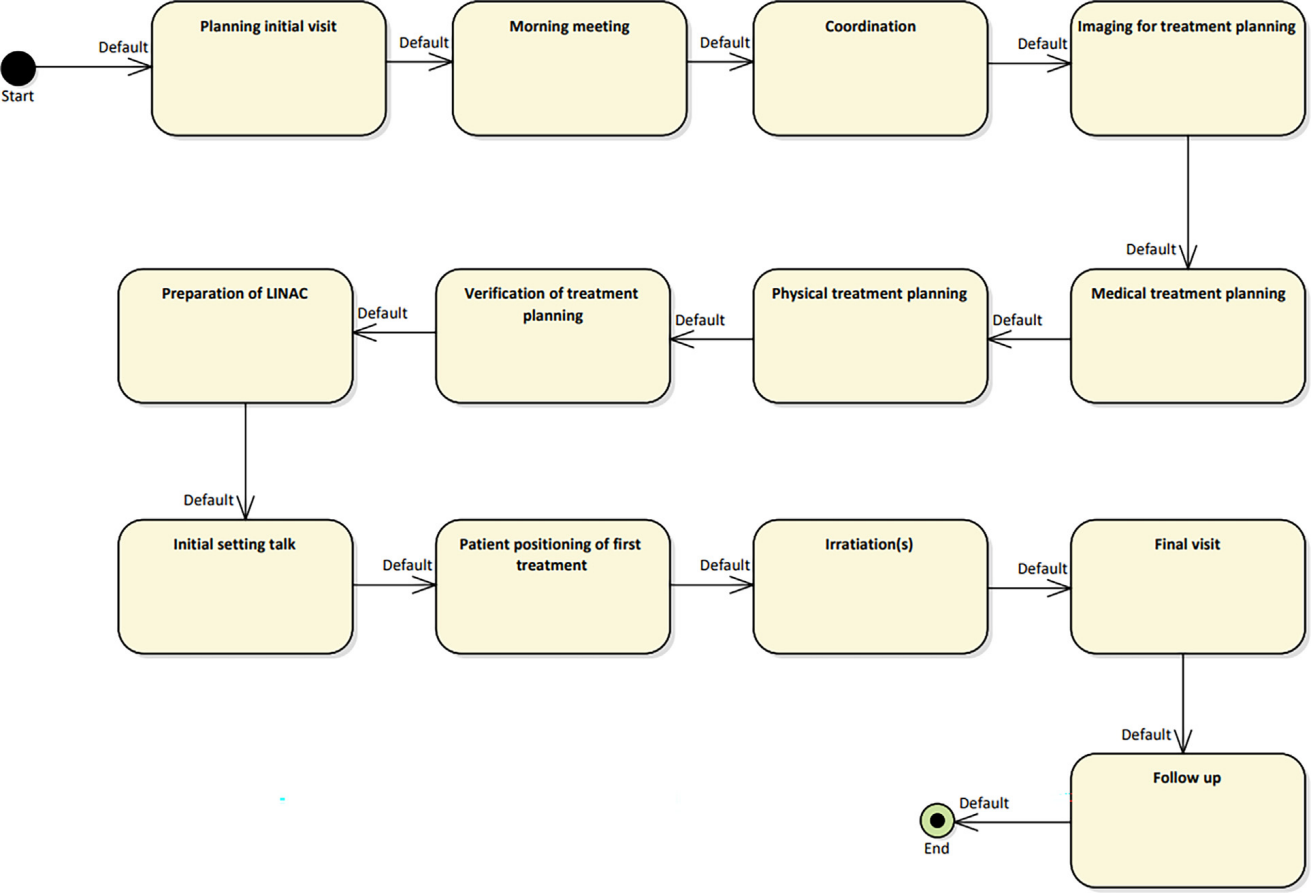
Overview of the main therapy level of the workflow. All steps of the workflow are shown in the top level.

In the **main therapy level**, all main workflow steps ${\mathrm{MWS}}_{i}=\left\{{\mathrm{MWS}}_{1},{\mathrm{MWS}}_{2},\ldots \right\}$ are summarized, which are identical for almost every workflow of individual patient treatments. These include imaging, treatment planning, treatment delivery, i.e., the coarse description of a radiation oncology workflow. Fig. 2 shows the currently implemented main therapy level of our clinic.

At the **care-path level**, a distinction is made between treatment methods and/or indications ${\mathrm{TM}}_{i}=\left\{{\mathrm{TM}}_{1},{\mathrm{TM}}_{2},\ldots \right\}$. For example, dedicated care paths could be whole brain rt, or mask for all patients treated with mask-based immobilization.

In addition to an activity in the care path level, also arrows are needed to indicate the workflow of a care-path. It should be noted that for an individual patient multiple care-paths/arrows can be assigned in parallel (e.g., mask and glioblastoma multiforme). Apart from specific workflows a default workflow exists including the corresponding care path activity (see Fig. 1). The default workflow is chosen if no activities exist for a specific workflow.

On the **procedure level** medical procedures $P_{i}=\left\{P_{1},P_{2},\ldots \right\}$ of a care-path are listed, such as acquiring a CT with contrast agent. Often such procedures require a (medical) device which is thus incorporated on the **device level** of the swim-lane diagram ${\mathrm{MD}}_{i}=\left\{{\mathrm{MD}}_{1},{\mathrm{MD}}_{2},\ldots \right\}$. For devices, the corresponding activity stores attributes of the device, e.g., IP address, or contact person (see Table 1 for all details).

**Table 1:** Attributes stored for medical devices and risks. The lists are not exhaustive but rather serve as the currently considered set. The final choice is up to the user.

Medical devices	Risks
IP-address	Severity (S)
MAC-address	Occurrence (O)
Contact person	Detectability (D)
Location	URL of risk entry in JIRA
Manufacturer	Corresponding SOP
Serial number	URL of measures
Inventory number	
User manual	
List of instructed personal	

Risks $R_{i}=\left\{R_{1},R_{2},\ldots \right\}$ are marked with a green box, can occur in any level, and can be linked to more than one activity (but not arrows). Further parameters associated to risks are listed Table 1 and Section ‘Implementation of data and risk analysis’.

Each element of the activity diagram has a unique ID. Activities can occur in different levels of the swim-lane diagram and multiple activities can be combined in a single level. The decisive factor for this is the workflow determined by the arrow which connects multiple (unique) activities. Once the workflow reaches the deepest level of the nested diagram (e.g., activity ${\mathrm{MD}}_{i}$ in Fig. 1) it returns to the next higher activity in a recursive manner.

### Implementation of data and risk analysis

In the central system for risk analysis of the university hospital (initially JIRA, v8.14, Atlassian, London; currently migrated to roXtra, Göppingen, details in [3]), all information on risks, their assessment (such as the parameters severity, occurrence, and detectability for a FMEA) and the existing mitigation measures are stored.

Each risk entry has a unique ID and the parameters are accessible via an URL that is stored in the attributes of a risk entry in the activity diagram (see Table 1). For risk assessments, the modeled workflows and the data stored in JIRA can thus easily be merged in a workflow specific manner.

**Figure 3:**
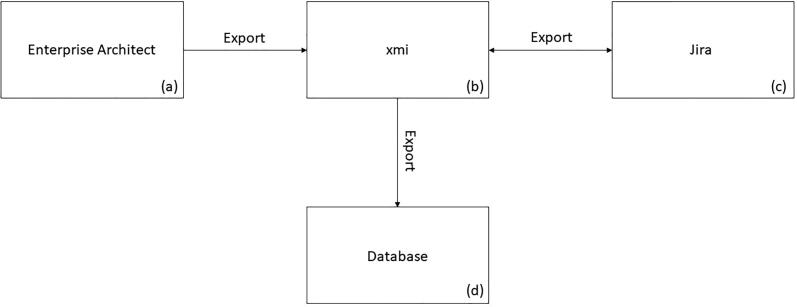
Software architecture to read out the XMI from the model. (a) Enterprise Architect: Software to model the workflows as activity diagrams, (b) xmi: Format in which the modeled diagram is being exported, (c) JIRA: Platform in which all risks are being recorded, (d) Database: MySQL database which stores the data from the xmi in a structured format.

For such assessments, the created model can be exported as an extensible markup language metadata interchange (xmi) file, Fig. 3. This is a machine-readable format that enables the exchange of UML models across platforms. A self-developed Python program reads the model from the xmi file, adds the parameters of the linked risks from JIRA and exports the data to the database.

This results in several evaluation options using the SQL database language. Simple queries, such as listing the IP address of a medical device, can be executed via a web interface.

However, the main benefit is the workflow-based risk assessment including the associated devices: for a specified workflow (e.g., mask) the activity diagram is parsed from Start to End resulting in a list of all activities of the specific workflow and their associated risks. Thus, a workflow specific FMEA including calculation of risk matrix or risk priority number can be achieved.

### Modelling of typical workflows

To illustrate the potential of the methodology, excerpts of the workflows ct appointment, glioblastoma multiforme, and treatment in deep inspiration breath hold (dibh) are modeled in detail below. They are all embedded in the main workflow shown in Fig. 2.

These specific workflows were chosen since they allow to focus on device, care-path, procedure, and device level, respectively.

**CT appointment:** To perform a CT, the medical devices listed in Table 2 are required. The Mosaiq OIS is used to schedule the appointment, which is exported by the ESI using a scheduling information unsolicited message (HL7 SIU). The message is processed by the communication server resulting in an entry in the DICOM worklist and an order message (HL7 ORM) send to the PACS. At the day of CT imaging, the CT reads the entries from the DICOM worklist, scans the patient, and sends the reconstructed DICOM images to the PACS which acknowledges the order to the DICOM worklist.

**Table 2:** Equipment required to perform a CT scan. A detailed description of the tasks of the medical devices is given in the text.

Medical device	Task
SOMATOM go.Open Pro (Siemens, Erlangen)	CT image acquisition
Mosaiq (Elekta, Stockholm)	Oncology Information System (OIS)
External Systems Interface (ESI) (Elekta, Stockholm)	Part of Mosaiq OIS for communication with external Clinical Information Systems
Communication server	Data exchange hub of the central IT
DICOM Worklist Server	Provides CT and UPACS with a list of patients imported from Mosaiq via the HL7 interface
PACS (Synedra, Innsbruck)	Picture archiving and communication system (PACS) of the central IT

**Glioblastoma multiforme:** A glioblastoma is a malignant brain tumor for which CT imaging is performed with contrast medium. For this purpose, a contrast medium injector is required in addition to the above-mentioned equipment. Each patient receives an individual mask for immobilization during CT imaging and radiotherapy. In addition, MR imaging is required. For treatment planning, CT and MRI images are fused for transferring structure sets from MRI to CT.

**Deep Inspiration Breath Hold (DIBH):** DIBH is a treatment method used for treatment of (mainly left-sided) breast tumors that aims at reducing heart and lung toxicity of the treatment.

Patients are instructed to hold their breath in a deep inspiration prior to CT acquisition and radiation of treatment fields. At UK Erlangen, the breath hold level is controlled via surface guidance (SGRT, surface guided radiation therapy) [21]. In addition to the CT in inspiration, another CT is acquired with the patient free-breathing, which is needed for SGRT-based patient positioning.

## Results

### CT appointment

**Figure 4:**
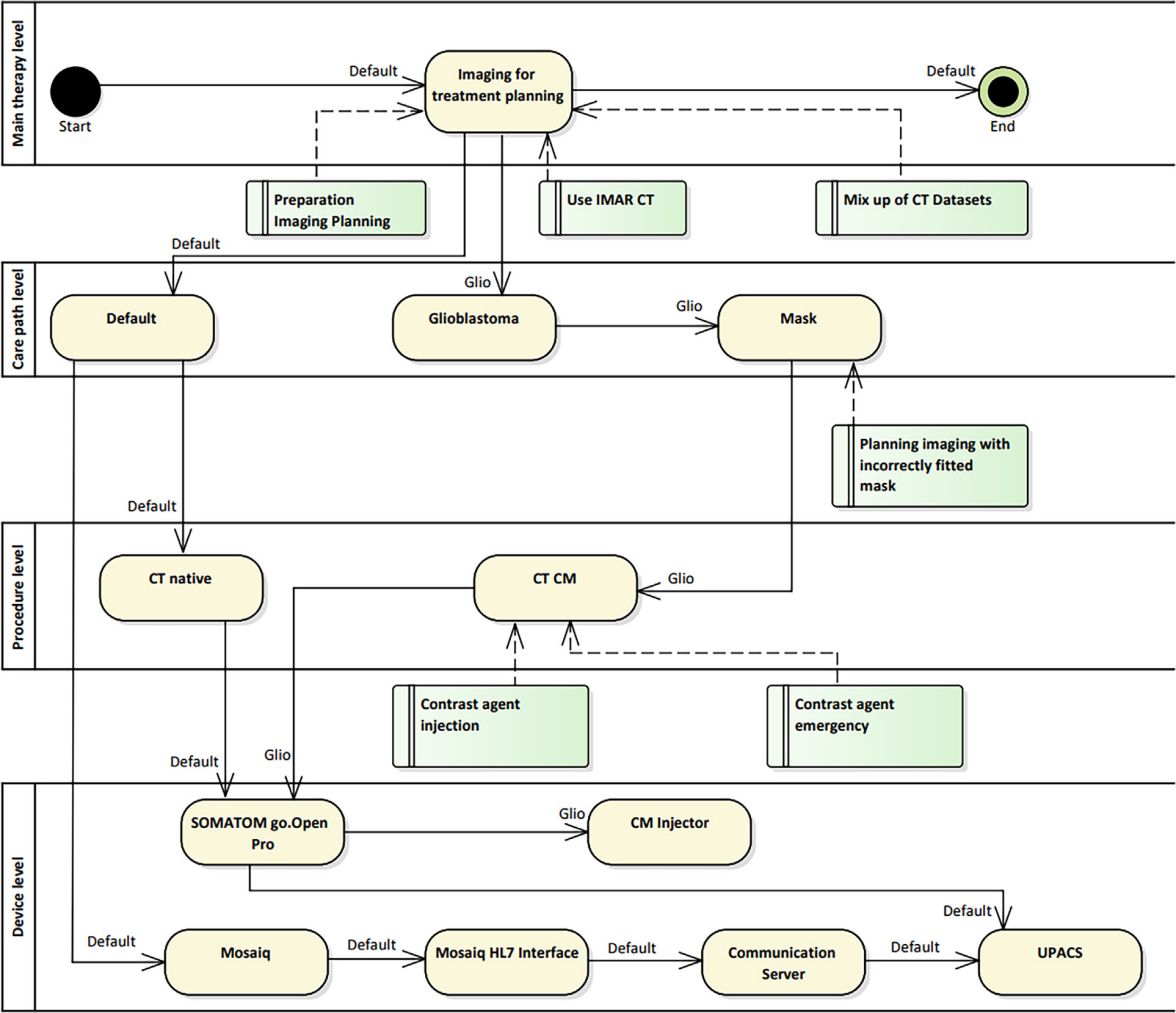
Section of the overall workflow for the imaging for treatment planning step with the workflow default and two sample workflows. The different levels of the swim lane are shown on the left. The figure reads from top to bottom and from left to right. The yellow boxes are workflow steps, the green boxes are risks that belong to the workflow steps. Note, that once the workflow reaches the deepest level of the nested diagram it recursively returns to the next higher activity.

Fig. 4 shows an overview of the workflows and medical devices which are used for a CT scan. Two main medical devices are used for imaging, the Siemens SOMATOM go.Open Pro and the UPACS. The risks linked to the top level apply to all workflows and the lower-level risks apply to specific workflows such as the workflow glioblastoma multiforme.

Depending on the workflow, a CT can be performed natively as well as with contrast medium. In the following, we will focus on the equipment that is necessary to perform a CT.

### Glioblastoma multiforme

**Figure 5:**
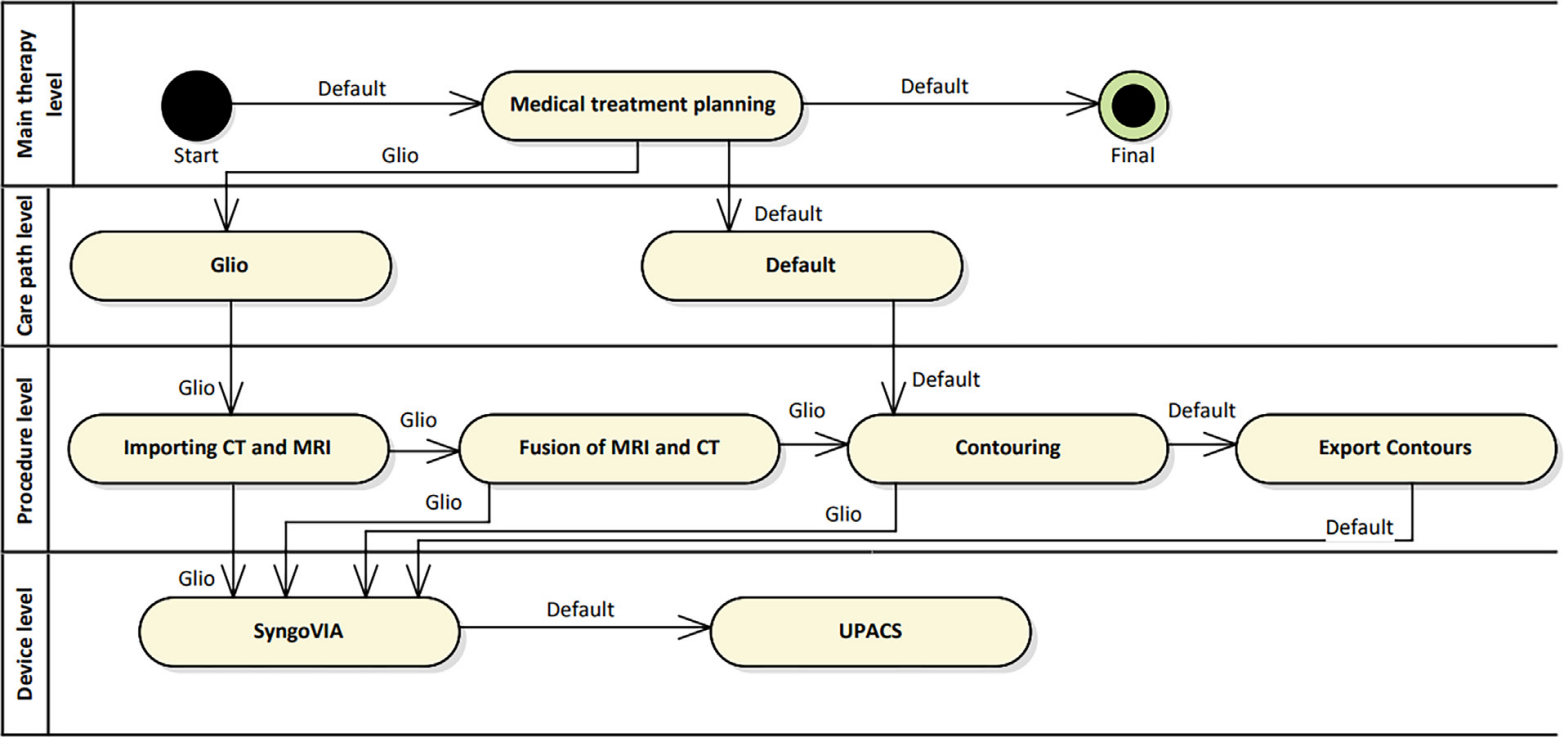
Step of medical treatment planning for workflows default and glioblastoma multiforme. Notation is adopted from [3].

Fig. 5 shows the medical treatment planning steps for the workflow glioblastoma multiforme as well as the default workflow. Import, fusion of MRI and CT, and contouring are performed in SyngoVIA (Siemens, Erlangen). Contouring and exporting the contours is the same for all workflows and thus summarized under the workflow default. Once contouring is complete, SyngoVIA exports its data to UPACS. From here, the data can be retrieved by the planning system.

### DIBH

**Figure 6:**
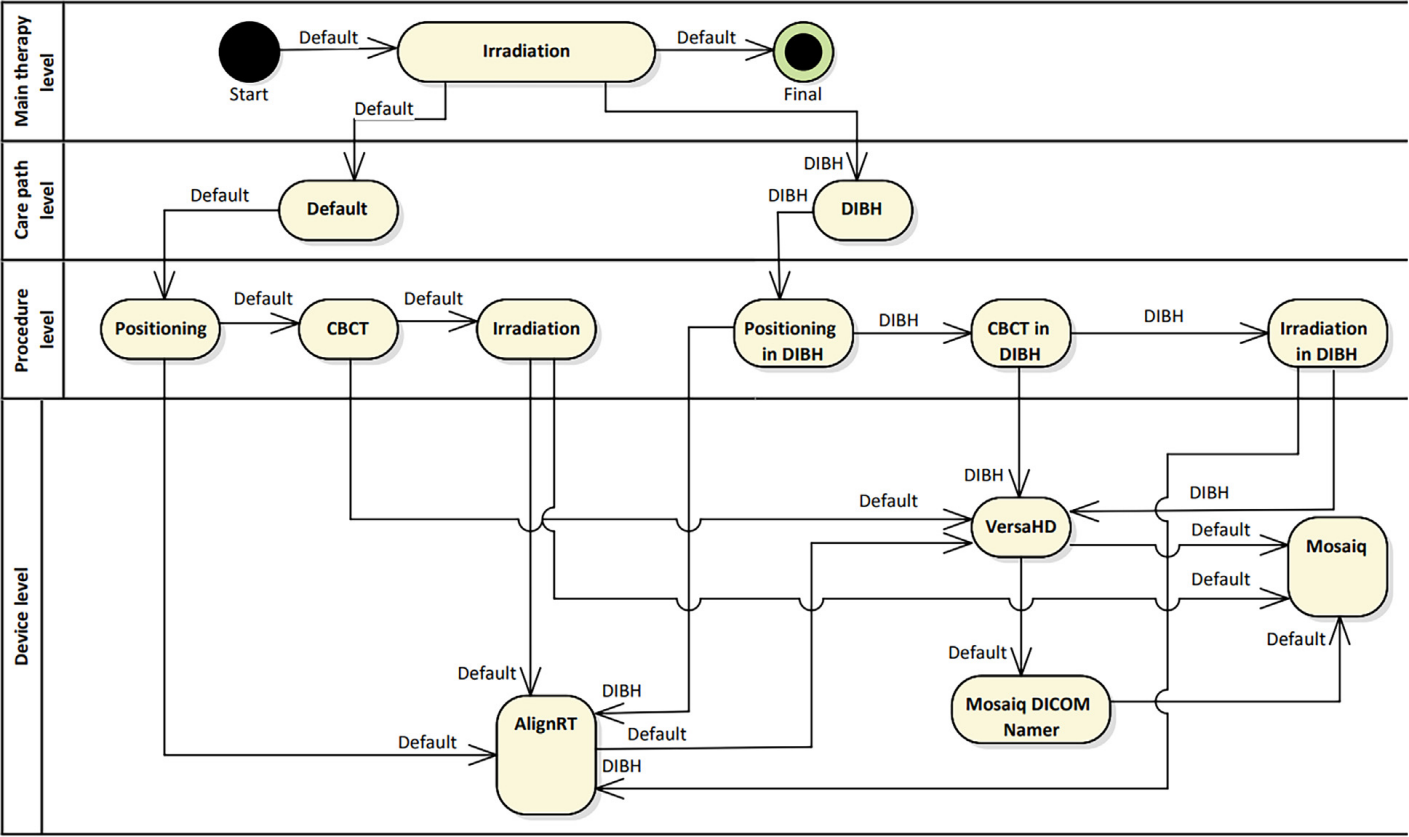
Workflow and equipment used for the workflows DIBH and default in the step of irradiating a patient. All steps on the Procedure level are performed in inspiration in case of workflow DIBH. Note, that once the workflow reaches the deepest level of the nested diagram it recursively returns to the next higher activity.

Fig. 6 shows the section of a patient's treatment within workflows and default. The dibh workflow differs from the default workflow in that all steps are performed in inspiration controlled by SGRT, requiring changes on the procedure level. The AlignRT system has a direct connection to the linear accelerator via the ICOM interface (Elekta, Stockholm). This allows to interrupt treatment if the breathing position is not maintained. For irradiation, the VersaHD accelerator and the Mosaiq OIS are required.

CBCT imaging data is sent from the VersaHD to the Mosaiq DICOM Namer (Elekta, Stockholm), which forwards it to the Mosaiq OIS.

### Workflow specific risk analysis

**Figure 7:**
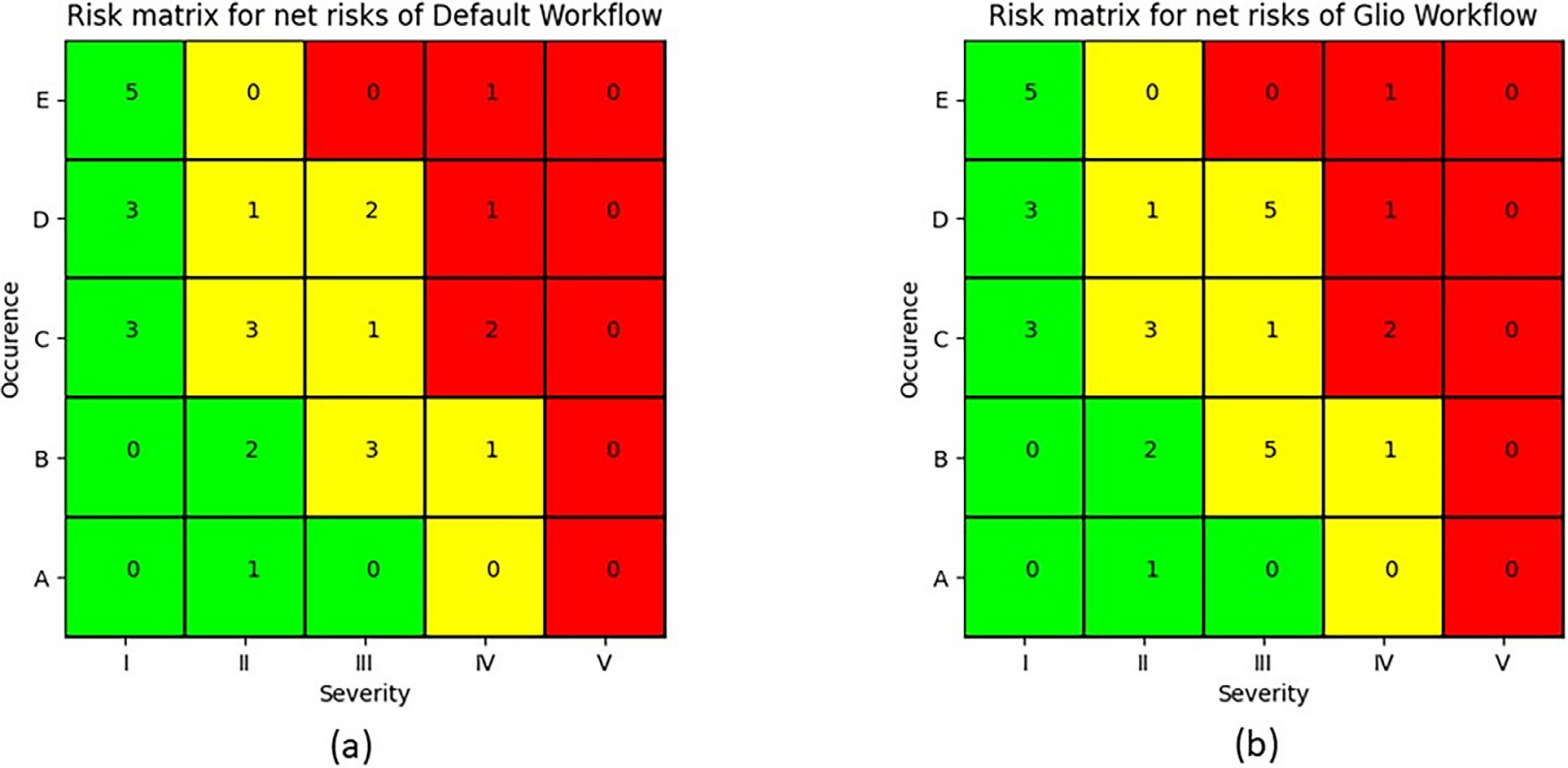
Risk matrix, for the workflow default (a) and workflow glioblastoma multiforme (b). Shown is the Occurrence and Severity for the net risk assessment (i.e. after implementation of measures – see [3] for details).

Fig. 7 shows the risk matrices of workflows glioblastoma multiforme and default. Fig. 4 shows that the risks on the main therapy level are collected first, followed by the risks on the care path and procedure level. Here, workflow default and glioblastoma multiforme differ since risks related to mask and contrast agent are not required per default. In summary, workflow glioblastoma multiforme inherits all risks related to the default plus 5 additional ones (related to contrast media, masks, and MRI) in categories (D, III) and (B, III). Category (E, IV) and (C, IV) risks are currently still mitigated by measures that have not been fully implemented.

## Discussion and conclusions

A methodology was presented to model the individual steps of a treatment workflow and the associated risks in an activity diagram. Different levels are used to create a link between the medical device level and the level of the main therapeutic workflows. All information about the workflow steps, the associated risks and the medical devices supporting the workflow are available in a database. With the help of the methodology, it is then possible to assess the complexity of workflows, to create individual risk matrices for each workflow and to find out which medical device occurs in which workflow. In addition, the model supports introducing new treatment methods or new medical devices into the workflow.

Many methods are currently used in radiation therapy to implement risk analysis using a FMEA [9], [22]. Most publications on risk analysis in radiotherapy refer to individual treatment methods [16], [23], [24] or specific steps of the treatment planning process [25], [26]. This is reasonable, since most errors occur during treatment planning [27] and physicists have a high workload in the entire therapy process [28]. For example Munbodh et al. reported on graph-based risk assessment with respect to pretreatment physics chart review [17]. They used a directed graph to decompose the chart review into individual steps which were associated with variables.

By analyzing the entry of variables, inconsistencies could be determined allowing an automated chart review but also risk assessment.

Their methodology is even more detailed than the one proposed in this manuscript but also focused on a niche of the whole treatment workflow (chart review) rather than the global view addressed here. Addressing all steps of a treatment with the detailed description proposed by Munbodh et al. would likely be not feasible for a typical clinic.

The presented methodology allows a standardized view of different workflows and risks covering the complete therapy process. It can be linked to any risk analysis software allowing unique links and automated read-out by an external software. This initially requires substantial effort for documenting the existing work processes in graphical form within the UML-capable software such as Enterprise Architect.

Once a graphical model of the workflows has been created, individual workflows can be readily changed, and complex workflows can be understood much easier than in many different text documents or spreadsheets that frequently are used for risk documentations.

In addition, the documentation includes medical devices. The risk analysis can thus be extended according to IEC 80001-1 readily [2]. Also, other necessities of a radiation oncology workflow such as immobilization equipment, or even medication can be covered if the model is extended accordingly.

Risk analysis in the proposed framework is currently limited to FMEA. Another frequently proposed methodology is a Fault Tree Analysis (FTA) [29] which is not (yet) implemented. However, the modeled workflows can serve as a good basis here. To further support the Fault Tree Analysis, active error reporting by the employees [30] or the medical devices themselves would be desirable. This could also be used to quantitatively check the effectiveness of (new) measures.

## Declaration of Competing Interest

The authors declare that they have no known competing financial interests or personal relationships that could have appeared to influence the work reported in this paper.

## References

[1] Moorman B. (2010). Medical Device Interoperability: Overview of Key Initiatives. Biomed Instrum Technol.

[2] DIN EN 80001-1:2011-11: Anwendung des Risikomanagements für IT-Netzwerke, die Medizinprodukte beinhalten.

[3] Lohmann D., Lang-Welzenbach M., Feldberger L., Sommer E., Bücken S., Lotter M., Ott O., Fietkau R., Bert C. (2022). Risk analysis for radiation therapy at UK Erlangen. Z Med Phys.

[4] Stamatis D.H. (2003).

[5] Ford E.C., Gaudette R., Myers L., Vanderver B., Engineer L., Zellars R. (2009). Evaluation of safety in a radiation oncology setting using failure mode and effects analysis. Int J Radiat Oncol Biol Phys.

[6] Thornton E., Brook O., Mendiratta-Lala M., Hallett D., Kruskal J. (2011). Application of failure mode and effect analysis in a radiology department. Radiographics.

[7] Ford E.C., Smith K., Terezakis S., Croog V., Gollamudi S., Gage I. (2014). A streamlined failure mode and effects analysis. Med Phys.

[8] Sawant A., Dieterich S., Svatos M., Keall P. (2010). Failure mode and effect analysis-based quality assurance for dynamic MLC tracking systems. Med Phys.

[9] Baehr A., Oertel M., Kröger K., Eich H.T., Haverkamp U. (2020). Implementing a new scale for failure mode and effects analysis (FMEA) for risk analysis in a radiation oncology department. Strahlenther Onkol.

[10] Ford E.C., Smith K., Harris K., Terezakis S. (2012). Prevention of a wrong location misadministration through the use of an intradepartmental incident learning system. Med Phys.

[11] Clark B., Brown R., Ploquin J., Kind A., Grimard L. (2010). The management ofradiation treatment error through incident learning. Radiother Oncol.

[12] Ford E.C., Evans S.B. (2018). Incident learning in radiation oncology: A review. Med Phys.

[13] Ford E.C., Evans S. (2012). Consensus recommendations for incident learning database structures in radiation oncology. Med Phys.

[14] Yang F., Cao N., Young L., Howard J., Logan W., Arbuckle T. (2015). Validating FMEA output against incident learning data: A study in stereotactic body radiation therapy. Med Phys.

[15] Hoisak J.D.P., Manger R., Dragojević I. (2021). Benchmarking failure mode and effects analysis of electronic brachytherapy with data from incident learning systems. Brachytherapy.

[16] Guckenberger M., Baus W., Blanck O., Combs S., Debus J., Engenhart C.R. (2020). Definition and quality requirements for stereotactic radiotherapy: consensus statement from the DEGRO/DGMP Working Group Stereotactic Radiotherapy and Radiosurgery. Strahlenther Onkol.

[17] Mundbodh R., Bowles J., Zaveri H. (2021). Graph-based risk assessment and error detection in radiation therapy. Med Phys.

[18] Baltzer H. (2011).

[19] Object Management Group, Unified Modeling Language. 2017 https://www.omg.org/spec/UML/2.5.1/About-UML/ (accessed 11 February 2022).

[20] Lucidchart Inc. 2022, What is a Swimlane Diagram? https://www.lucidchart.com/pages/tutorial/swimlane-diagram (accessed 11 February 2022).

[21] Freislederer P., Kügele M., Öllers M., Swinnen A., Bert C. (2020). Recent advanced in Surface Guided Radiation Therapy. Radiat Oncol.

[22] Huq M.S., Fraass B., Dunscombe P., Gibbons J., Ibbott G., Mundt A. (2016). The report of Task Group 100 of the AAPM: Application of risk analysis methods to radiation therapy quality management. Med Phys.

[23] Shariff M., Stillkrieg W., Lotter M., Lohmann D., Weissmann T., Fietkau R., Bert C. (2021). Dosimetry, Optimization and FMEA of Total Skin Electron Irradiation (TSEI). Z Med Phys.

[24] Perks J., Stanic S., Stern R., Henk B., Nelson M., Harse R. (2012). Failure mode and effect analysis for delivery of lung stereotactic body radiation therapy. Int J Radiat Oncol Biol Phys.

[25] Covington E., Chen X., Younge K., Lee C., Matuszak M., Kessler M. (2016). Improving treatment plan evaluation with automation. J Appl Clin Med Phys.

[26] Liu S., Bush K., Bertini J., Fu Y., Lewis J., Pham D. (2019). Optimizing efficiency and safety in external beam radiotherapy using automated plan check (APC) tool and six sigma methodology. J Appl Clin Med Phys.

[27] Gopan O., Zeng J., Novak A., Nyflot M., Ford E. (2016). The effectiveness of pretreatment physics plan review for detecting errors in radiation therapy. Medical Phys.

[28] Mazur L., Mosaly P., Jackson M., Chang S., Burkhardt K., Adams R. (2012). Quantitative assessment of workload and stressors in clinical radiation oncology. Int J Radiat Oncol Biol Phys.

[29] Rath F. (2008). Tools for developing a quality management program: proactive tools (process mapping, value stream mapping, fault tree analysis, and failure mode and effects analysis). Int J Radiation Oncology Biol Phys.

[30] Kessels-Habraken M., Van der Schaaf T., De Jonge J., Rutte C., Kekvliet K. (2009). Integration of prospective and retrospective methods for risk analysis in hospitals. Int J Quality Health Care.

